# Iron serum levels and iron homeostasis parameters in patients with nosocomial pneumonia treated with cefiderocol: post hoc analysis of the APEKS-NP study

**DOI:** 10.1007/s10096-021-04399-9

**Published:** 2022-01-13

**Authors:** Eric P. Skaar, Roger Echols, Yuko Matsunaga, Anju Menon, Simon Portsmouth

**Affiliations:** 1 Department of Pathology, Microbiology, and Immunology, Vanderbilt University Medical Center, Nashville, TN 37232 USA; 2 Infectious Disease Drug Development Consulting, LLC, Easton, CT 06612 USA; 3 Shionogi Inc., 300 Campus Drive, Florham Park, NJ 07932 USA

**Keywords:** Cefiderocol, Critically ill, Iron homeostasis, Nosocomial pneumonia, Safety

## Abstract

Critically ill patients often present with low serum iron levels or anemia. We evaluated the impact of iron levels and iron homeostasis on the efficacy and safety of cefiderocol, an iron-chelator siderophore cephalosporin, in patients with nosocomial pneumonia in a post hoc analysis of the randomized, double-blind, Phase 3 APEKS-NP study (NCT03032380). Patients with Gram-negative nosocomial pneumonia received cefiderocol 2 g, 3-h infusion, q8h, or high-dose, extended-infusion meropenem 2 g, 3-h infusion, q8h, for 7–14 days. Efficacy and safety parameters, including specific iron homeostasis parameters (i.e., hepcidin, iron, total iron binding capacity, transferrin saturation), were analyzed according to baseline iron levels. In the cefiderocol and meropenem arms, 79.1% (117/148) and 83.3% (125/150) randomized patients, respectively, had low baseline serum iron levels. Rates of 14-day (12.3% [14/114] vs 11.6% [14/121]) and 28-day all-cause mortality (20.5% [23/112] vs 19.0% [23/121]), clinical cure (63.2% [72/114] vs 67.2% [82/122]), and microbiological eradication (43.6% [41/94] vs 48.1% [51/106]) at test of cure were similar in cefiderocol vs meropenem arms, respectively. In the overall safety population, rates of anemia-related adverse events were similar (cefiderocol arm 18.2% [27/148], meropenem arm 18.7% [28/150]). Changes from baseline to test of cure in hepcidin, iron, total iron binding capacity, and transferrin saturation were similar between treatment arms. Cefiderocol treatment did not affect iron homeostasis, and its efficacy and safety were not influenced by baseline serum iron levels. Clinicaltrials.gov registration: NCT03032380. Date of registration: 26 January 2017.

**Supplementary Information:**

The online version contains supplementary material available at 10.1007/s10096-021-04399-9.

## Introduction

Iron is an essential nutrient for both humans under physiological conditions and for pathogenic bacteria during infection [1–3]. Strict regulation of iron homeostasis in humans under both physiological and pathological conditions is essential [1, 3, 4]. Under normal physiological conditions, iron is mainly stored intracellularly in erythrocytes, or is bound in the plasma to transferrin, which has a high affinity for ferric iron (Fe^3+^) [1, 3].

Following an insult, such as an infection, iron homeostasis is disrupted, and nutritional immunity is enhanced [1, 4]. Nutritional immunity is a vertebrate strategy to restrict the availability of essential nutrients, including iron, to invading pathogens [1, 2, 5, 6]. Hepcidin, a peptide hormone made in the liver [7], has a major role in eliciting the hypoferremic response to infection [1, 7, 8]. During inflammation and infection, increased hepcidin levels, in response to increased levels of inflammatory molecules such as IL-6, can decrease serum iron levels to a degree that is consistent with iron deficiency or anemia [8]. Additionally, hepcidin can lead to a reduction in serum transferrin iron saturation [4]. Reduced iron availability at the infection site has the potential to decrease growth and virulence of pathogenic bacteria [1].

One approach Gram-negative bacteria have evolved to overcome nutritional immunity is the secretion of siderophore molecules, which have a higher Fe^3+^-binding affinity than transferrin. The siderophore–iron complexes are captured by siderophore receptors, allowing bacteria to import iron [2, 3] across the outer membrane.

Siderophores have been exploited in the drive to find new, more effective antibiotics against Gram-negative pathogens, by taking advantage of siderophore uptake as a method of delivering antibiotics into the bacterial cell in a “Trojan horse” strategy [3, 6, 9]. Cefiderocol is an iron-chelator, siderophore cephalosporin with broad activity against aerobic Gram-negative bacteria, including Enterobacterales and the non-fermenter species *Acinetobacter baumannii*, *Pseudomonas aeruginosa*, and *Stenotrophomonas maltophilia* [10]. Cefiderocol has demonstrated efficacy in the treatment of patients with complicated urinary tract infections (cUTI; APEKS-cUTI [11]), in patients with infections of various origin caused by carbapenem-resistant Gram-negative pathogens (CREDIBLE-CR [12]), and in patients with nosocomial pneumonia (APEKS-NP [13]).

Given that the transport of cefiderocol exploits bacterial iron acquisition systems, it is important to establish whether conditions associated with iron deficiency or iron overload could have any impact on its efficacy. This post hoc analysis of the Phase 3 APEKS-NP study, which demonstrated that cefiderocol was non-inferior for 14-day all-cause mortality (ACM) to high-dose, extended-infusion meropenem in critically ill patients with Gram-negative nosocomial pneumonia [13], explored the impact of serum baseline iron levels and iron supplementation on the relative efficacy of cefiderocol and meropenem.

## Material and methods

### Study design

The methodology for the APEKS-NP study has previously been described in detail [13]. The study design, ethics, inclusion and exclusion criteria, treatment allocation, patient populations, and outcome definitions are described by Wunderink et al. [13]. The study protocol was approved by relevant institutional review boards and independent ethics committees. All patients or their legal guardians provided written informed consent [13].

In brief, APEKS-NP (clinicaltrials.gov NCT03032380) was an international, multicenter, randomized (1:1), double-blind, non-inferiority Phase 3 study in adult patients (≥ 18 years of age) with Gram-negative nosocomial pneumonia (hospital-acquired pneumonia, ventilator-associated pneumonia, and healthcare-associated pneumonia); the study compared the efficacy and safety of cefiderocol treatment vs high-dose, extended-infusion meropenem treatment. Patients with suspected or confirmed nosocomial pneumonia caused by a Gram-negative pathogen based on Gram-stain of an appropriate respiratory sample and findings of chest X-ray or computed tomography imaging were eligible for inclusion. Patients were not eligible for enrollment if they had pneumonia caused by a pathogen known at the time of randomization to be carbapenem resistant, an Acute Physiology and Chronic Health Evaluation II (APACHE II) score > 35, chemical or aspiration pneumonia, cystic fibrosis, bronchiectasis, or mold infections. Detailed inclusion and exclusion criteria are described by Wunderink et al. [13].

Patients were randomly allocated to receive either cefiderocol (2 g, q8h, 3-h infusion) or high-dose, extended-infusion meropenem (2 g, q8h, 3-h infusion) for 7–14 days. Dose adjustment according to renal function, including augmented renal clearance, was described elsewhere [13]. The duration of treatment could be extended at the discretion of the investigator to 21 days. No adjunctive Gram-negative therapy was allowed. Linezolid was administered for at least 5 days in both arms for coverage of methicillin-resistant *Staphylococcus aureus* and/or other Gram-positive bacteria.

### Outcomes

The primary endpoint was day 14 ACM in the modified intention-to-treat (mITT) population (including all patients receiving ≥ 1 dose of study drug but excluding those with monomicrobial Gram-positive pathogens). The secondary endpoints included clinical and microbiological outcomes at test of cure (TOC; end of treatment [EOT] + 7 [± 2] days, where EOT was the last day of study treatment) in the mITT population. Safety was investigated through to end of study (EOS; EOT + 28 [± 3] days) in the safety population, which included all patients who received at least one dose of study drug.

In this post hoc analysis, ACM at days 14 and 28 and EOS, and clinical and microbiological outcomes at EOT, TOC, and follow-up (EOT + 14 [± 3] days) in the mITT population were analyzed according to baseline total serum iron levels, which were sampled as required per protocol and analyzed at central laboratory with centrally defined normal ranges. ACM rates were calculated for the number of patients with known vital status at days 14 and 28 and at EOS. The microbiological eradication rates were calculated for the number of patients with confirmed respiratory Gram-negative pathogen at baseline.

As part of the safety assessment, specialized laboratory tests (Supplementary Table 1) including measurements of hepcidin, total serum iron level (i.e., low [male: < 11 µmol/L; female: < 7 µmol/L] and normal [male: 11–32 µmol/L; female: 7–31 µmol/L] iron levels), transferrin iron saturation, and total iron binding capacity (TIBC) were carried out at baseline and TOC, and changes between these two time points were analyzed in the safety population. Hemoglobin and hematocrit levels were assessed as part of routine laboratory investigation at screening, early assessment (days 3–4), EOT, TOC, and follow-up, and changes between baseline and TOC were analyzed in the safety population. In the subgroup of patients who received supplementation to correct anemia (i.e., blood transfusion, iron supplementation, or both) by EOT, ACM and clinical and microbiological outcomes at TOC (mITT population) and baseline-to-TOC changes in iron parameters (safety population) were evaluated. Patients were grouped and analyzed according to the supplementations they received or not received: (1) patients with blood transfusion only by EOT vs patients without blood transfusion; (2) patients with iron supplementation only by EOT vs patients without iron supplementation; and (3) patients with blood transfusion and/or iron supplementation by EOT vs patients without any supplementation. In the third subgroup, patients could have received both blood transfusion and iron supplementation at any time before EOT.

### Statistical analyses

The intention-to-treat (ITT) population comprised all patients who received at least one dose of study drug. The mITT population comprised all patients who received at least one dose of study drug and had a suspected or confirmed respiratory Gram-negative pathogen at randomization. The safety population comprised all patients who received at least one dose of study drug and were assessed for the actual treatment received. The subgroup analyses were performed in the ITT, mITT, and safety populations in the subgroups of patients with available baseline iron levels (i.e., low and normal iron levels), and available information on administration of supplementations (i.e., blood transfusion, iron supplementation, or both) regardless of baseline iron levels.

Treatment differences for ACM and clinical and microbiological outcomes were expressed as the effect of cefiderocol minus that of meropenem, and two-sided 95% confidence intervals (CIs) were calculated using a normal approximation to the difference between the two binomial proportions (Wald method). Analysis of ACM was performed for all patients with known vital status. Continuous variables are summarized using the number of non-missing observations, the arithmetic mean and standard deviation (SD) as summary statistics. Categorical variables are summarized by using the frequency count and percentage of patients in each category. Missing data were not replaced or imputed. SAS version 9.2 or higher (SAS Institute, Cary, NC, USA) was used for all analyses.

## Results

### Patient characteristics

Overall, 298 patients were randomized in the ITT/safety population in the APEKS-NP study. Data on baseline serum iron concentrations (“iron levels”) were available for 292 patients in the ITT population, of whom 242 (82.9%) had low iron levels (cefiderocol arm *n* = 117, meropenem arm *n* = 125) and 50 (17.1%) had normal iron levels (cefiderocol arm *n* = 27, meropenem arm *n* = 23) (Table 1). The patient characteristics in the overall ITT/safety population have been described elsewhere [13].

**Table 1:** Demographics and baseline clinical characteristics and receipt of iron supplementation by end of treatment according to baseline iron level category: intention-to-treat/safety population (patients with known iron levels were included)

	Low iron level		Normal iron level	
	Cefiderocol (*N* = 117)	Meropenem (*N* = 125)	Cefiderocol (*N* = 27)	Meropenem (*N* = 23)
Age, mean (SD), y	64.2 (14.7)	65.5 (15.5)	66.6 (13.9)	65.7 (12.9)
Sex, male, no. (%)	88 (75.2)	93 (74.4)	10 (37.0)	9 (39.1)
BMI, mean (SD), kg/m^2^	26.1 (5.4)	26.7 (7.0)	27.6 (8.6)	26.2 (6.0)
Region, no. (%)				
North America	1 (0.9)	4 (3.2)	4 (14.8)	2 (8.7)
Europe	80 (68.4)	85 (68.0)	17 (63.0)	13 (56.5)
Asia–Pacific	36 (30.8)	36 (28.8)	6 (22.2)	8 (34.8)
Race, no. (%)				
White	78 (66.7)	84 (67.2)	21 (77.8)	14 (60.9)
Black or African American	0	1 (0.8)	0	0
Asian	37 (31.6)	36 (28.8)	6 (22.2)	8 (34.8)
Other	2 (1.7)	3 (2.4)	0	1 (4.3)
Missing	0	1 (0.8)	0	0
Clinical diagnosis, no. (%)				
VAP	50 (42.7)	58 (46.4)	9 (33.3)	6 (26.1)
HAP	48 (41.0)	47 (37.6)	10 (37.0)	13 (56.5)
HCAP	19 (16.2)	20 (16.0)	8 (29.6)	4 (17.4)
Ventilated at randomization, no. (%)	77 (65.8)	76 (60.8)	12 (44.4)	9 (39.1)
Creatinine clearance				
Mean (SD), mL/min	78.2 (57.1)	84.5 (59.7)	76.8 (50.7)	67.0 (24.3)
Median (min, max), mL/min	64.0 (5, 306)	69.5 (7, 281)	67.4 (5, 267)	67.7 (24, 117)
> 120 mL/min, no. (%)	18 (15.4)	25 (20.0)	4 (14.8)	0
> 80–120 mL/min, no. (%)	26 (22.2)	29 (23.2)	6 (22.2)	6 (26.1)
> 50–80 mL/min, no. (%)	32 (27.4)	25 (20.0)	9 (33.3)	12 (52.2)
30–50 mL/min, no. (%)	24 (20.5)	28 (22.4)	5 (18.5)	3 (13.0)
< 30 mL/min, no. (%)	17 (14.5)	18 (14.4)	3 (11.1)	2 (8.7)
Empiric treatment failure, no. (%)	42 (35.9)	35 (28.0)	7 (25.9)	12 (52.2)
APACHE II score				
Mean (SD)	16.3 (6.2)	16.7 (6.6)	15.1 (6.0)	14.6 (7.5)
Median (min, max)	16.0 (3, 34)	16.0 (4, 35)	14.0 (8, 33)	12.0 (4, 32)
CPIS (ventilated)	*N* = 77	*N* = 76	*N* = 12	*N* = 9
Mean (SD)	5.8 (1.7)	5.9 (1.9)	6.3 (1.1)	5.3 (1.3)
Median (min, max)	6.0 (2, 10)	6.0 (3, 10)	6.0 (4, 8)	5.0 (3, 7)
SOFA score (ventilated)	*N* = 77	*N* = 76	*N* = 12	*N* = 8
Mean (SD)	6.1 (2.8)	6.3 (3.2)	5.7 (2.6)	6.6 (2.7)
Median (min, max)	6.0 (1, 13)	6.0 (0, 16)	5.0 (3, 11)	6.0 (4, 12)
ICU admission, no. (%)	88 (75.2)	86 (68.8)	14 (51.9)	11 (47.8)
Medical history				
Blood and lymphatic system disorders, no. (%)	34 (29.1)	40 (32.0)	4 (14.8)	6 (26.1)
Anemia	23 (19.7)	23 (18.4)	4 (14.8)	4 (17.4)
Anemia macrocytic	1 (0.9)	2 (1.6)	0	0
Coagulation factor deficiency	1 (0.9)	1 (0.8)	0	0
Coagulopathy	1 (0.9)	1 (0.8)	0	2 (8.7)
Hemorrhagic anemia	2 (1.7)	4 (3.2)	0	0
Hypersplenism	0	1 (0.8)	0	0
Hypocoagulable state	1 (0.9)	0	0	0
Hypoprothrombinemia	1 (0.9)	0	0	0
Iron deficiency anemia	2 (1.7)	3 (2.4)	0	0
Leukopenia	0	1 (0.8)	0	0
Lymphopenia	0	1 (0.8)	0	0
Nephrogenic anemia	3 (2.6)	1 (0.8)	0	1 (4.3)
Normochromic normocytic anemia	0	1 (0.8)	0	0
Splenic lesion	0	0	0	1 (4.3)
Thrombocytopenia	3 (2.6)	9 (7.2)	0	1 (4.3)
Iron supplementation received by EOT, no. (%)				
Blood transfusion^a^	21 (17.9)	14 (11.2)	3 (11.1)	3 (13.0)
Iron supplementation	15 (12.8)	8 (6.4)	1 (3.8)	3 (13.0)
Blood transfusion and/or iron supplementation^a^	31 (26.5)	21 (16.8)	4 (14.9)	6 (26.1)

*BMI*, body mass index; *VAP*, ventilator-associated pneumonia; *HAP*, hospital-acquired pneumonia; *HCAP*, healthcare-associated pneumonia; *APACHE II*, Acute Physiology and Chronic Health Evaluation II; *CPIS*, Clinical Pulmonary Infection Score; *SOFA*, Sequential Organ Failure Assessment; *ICU*, intensive care unit; *EOT*, end of treatment; *SD*, standard deviation

^a^Number of patients with known baseline iron levels among those who received blood transfusion; for 2 patients, baseline iron level data were not available

In the subgroup of patients with available baseline iron levels, there were some numerical differences at baseline between low and normal iron level groups (Table 1), with low iron levels being more common among males (cefiderocol arm 75.2% vs 37.0%, meropenem arm 74.4% vs 39.1%, respectively), patients ventilated at randomization (cefiderocol arm 65.8% vs 44.4%, meropenem arm 60.8% vs 39.1%, respectively), and patients admitted to the intensive care unit (cefiderocol arm 75.2% vs 51.9%, meropenem arm 68.8% vs 47.8%, respectively). Median APACHE II scores were slightly higher among patients with low (cefiderocol arm 16.0, meropenem arm 16.0) compared with normal (cefiderocol arm 14.0, meropenem arm 12.0) iron levels (Table 1). However, for both low and normal iron level groups, patient characteristics were generally similar between treatment arms (Table 1). According to medical history data, approximately 15–20% of patients in each subgroup in the ITT/safety population had anemia of various origins at randomization (Table 1).

### ACM, clinical cure, and microbiological eradication

The mITT population, which excluded three patients in each arm who had only Gram-positive pathogens, with known baseline iron level comprised 286 patients (cefiderocol arm *n* = 141, meropenem arm *n* = 145). The primary endpoint in the study was ACM at day 14. Among patients with low iron levels, ACM at day 14 was 12.3% in the cefiderocol arm and 11.6% in the meropenem arm (treatment difference 0.7; 95% CI: − 7.6, 9.0). These rates were similar for patients with normal iron levels (Table 2).

**Table 2:** All-cause mortality rates according to baseline iron level category: modified intention-to-treat population

ACM rate, *n*/*N*′ (%)	Low iron level			Normal iron level		
	Cefiderocol	Meropenem	Difference (95% CI)	Cefiderocol	Meropenem	Difference (95% CI)
Day 14	14/114 (12.3)	14/121 (11.6)	0.7 (− 7.6, 9.0)	3/27 (11.1)	3/23 (13.0)	− 1.9 (− 20.1, 16.2)
Day 28	23/112 (20.5)	23/121 (19.0)	1.5 (− 8.7, 11.8)	6/27 (22.2)	5/23 (21.7)	0.5 (− 22.5, 23.5)
EOS	30/111 (27.0)	27/121 (22.3)	4.7 (− 6.4, 15.8)	6/27 (22.2)	5/23 (21.7)	0.5 (− 22.5, 23.5)

The percentage was calculated using number of patients (*N*′) with known vital status within each category as the denominator. Only patients with known baseline iron levels were included in the analysis

*ACM*, all-cause mortality; *EOS*, end of study; *CI*, confidence interval

ACM rates at day 28 and EOS were also similar between treatment arms in both subgroups of patients with low and normal baseline iron levels (Table 2). Clinical cure rates by patient at EOT, TOC, and follow-up were similar between treatment arms in patients with low baseline iron levels and in those with normal baseline iron levels (Table 3). A numerical difference in microbiological eradication rates was observed at TOC and follow-up in patients with normal baseline iron levels (TOC: cefiderocol arm 65.4%, meropenem arm 50.0%; treatment difference 15.4, 95% CI: − 13.2, 43.9; follow-up: cefiderocol arm 65.4%, meropenem arm 40.0%; treatment difference 25.4, 95% CI: − 2.8, 53.6). Clinical cure rates and microbiological eradication rates were generally numerically higher among patients with normal, compared with low, baseline iron levels (Table 3).

**Table 3:** Clinical and microbiological response rates according to baseline iron level category: modified intention-to-treat population

	Low iron level			Normal iron level		
	Cefiderocol	Meropenem	Difference (95% CI)	Cefiderocol	Meropenem	Difference (95% CI)
Clinical response rate, *n*/*N* (%)						
End of treatment						
Clinical cure	86/114 (75.4)	99/122 (81.1)	− 5.7 (− 16.2, 4.8)	22/27 (81.5)	20/23 (87.0)	− 5.5 (− 25.6, 14.6)
Clinical failure	19/114 (16.7)	18/122 (14.8)		2/27 (7.4)	2/23 (8.7)	
Indeterminate	9/114 (7.9)	5/122 (4.1)		3/27 (11.1)	1/23 (4.3)	
Test of cure						
Clinical cure	72/114 (63.2)	82/122 (67.2)	− 4.1 (− 16.2, 8.1)	19/27 (70.4)	16/23 (69.6)	0.8 (− 24.7, 26.3)
Clinical failure	24/114 (21.1)	25/122 (20.5)		3/27 (11.1)	5/23 (21.7)	
Indeterminate	18/114 (15.8)	15/122 (12.3)		5/27 (18.5)	2/23 (8.7)	
Follow-up						
Sustained clinical cure	63/114 (55.3)	71/122 (58.2)	− 2.9 (− 15.6, 9.7)	19/27 (70.4)	14/23 (60.9)	9.5 (− 16.9, 35.9)
Clinical failure	24/114 (21.1)	25/122 (20.5)		3/27 (11.1)	5/23 (21.7)	
Relapse	3/114 (2.6)	2/122 (1.6)		0	0	
Indeterminate	24/114 (21.1)	24/122 (19.7)		5/27 (18.5)	4/23 (17.4)	
Microbiological response rate, *n*/*N*′ (%)						
End of treatment						
Eradication	59/94 (62.8)	70/106 (66.0)	− 3.3 (− 16.6, 10.0)	19/26 (73.1)	14/20 (70.0)	3.1 (− 23.3, 29.4)
Persistence	13/94 (13.8)	15/106 (14.2)		3/26 (11.5)	4/20 (20.0)	
Indeterminate	22/94 (23.4)	21/106 (19.8)		4/26 (15.4)	2/20 (10.0)	
Test of cure						
Eradication	41/94 (43.6)	51/106 (48.1)	− 4.5 (− 18.3, 9.3)	17/26 (65.4)	10/20 (50.0)	15.4 (− 13.2, 43.9)
Persistence	20/94 (21.3)	22/106 (20.8)		3/26 (11.5)	5/20 (25.0)	
Indeterminate	33/94 (35.1)	33/106 (31.1)		6/26 (23.1)	5/20 (25.0)	
Follow-up						
Sustained eradication	36/94 (38.3)	41/106 (38.7)	− 0.4 (− 13.9, 13.1)	17/26 (65.4)	8/20 (40.0)	25.4 (− 2.8, 53.6)
Persistence	21/94 (22.3)	23/106 (21.7)		3/26 (11.5)	5/20 (25.0)	
Recurrence	0	2/106 (1.9)		1/26 (3.8)	0	
Indeterminate	37/94 (39.4)	40/106 (37.7)		5/26 (19.2)	7/20 (35.0)	

Only patients with known baseline iron levels were included in the analysis

*CI*, confidence interval; *ITT*, intention-to-treat; *N*, patients in the modified ITT population with the corresponding baseline iron category; *N′*, patients with non-missing baseline pathogens

### Safety parameters

In the overall ITT/safety population (*N* = 298) in the study, there was no difference between treatment arms in the frequency of adverse events related to anemia (Table 4).

**Table 4:** Adverse events related to anemia according to baseline iron level category: safety population

System organ class Preferred term, *n* (%)	Overall	
	Cefiderocol (*N* = 148)	Meropenem (*N* = 150)
Blood and lymphatic system disorders	27 (18.2)	28 (18.7)
Anemia	12 (8.1)	12 (8.0)
Anemia of chronic disease	2 (1.4)	0
Hemorrhagic anemia	0	2 (1.3)
Iron-deficiency anemia	3 (2.0)	0
Nephrogenic anemia	0	1 (0.7)
Normochromic normocytic anemia	1 (0.7)	0
Investigations	32 (21.6)	29 (19.3)
Hemoglobin decreased	1 (0.7)	0
Red blood cell count decreased	1 (0.7)	0

**Fig. 1:**
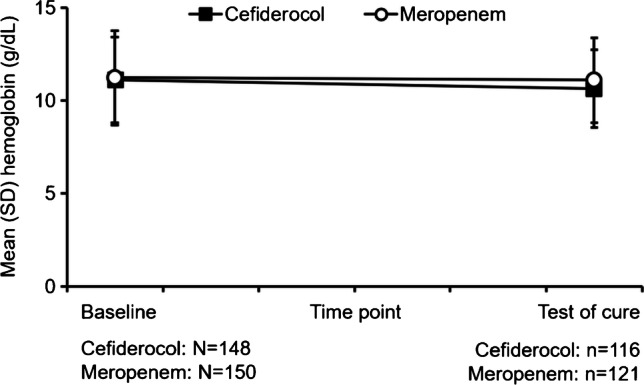
Changes in serum hemoglobin between baseline and test of cure in the overall safety population

Of these 298 patients, 237 patients (cefiderocol arm *n* = 116, meropenem arm *n* = 121) had hemoglobin measurements available at both baseline and TOC. A similar number of patients in each treatment arm (31/116 [26.7%] patients receiving cefiderocol and 30/121 [24.8%] receiving meropenem) had ≥ 1.5 g/dL reduction in hemoglobin between baseline and TOC. The course of changes in hemoglobin levels between baseline and TOC was similar in the cefiderocol and meropenem treatment arms (Fig. 1).

**Fig. 2:**
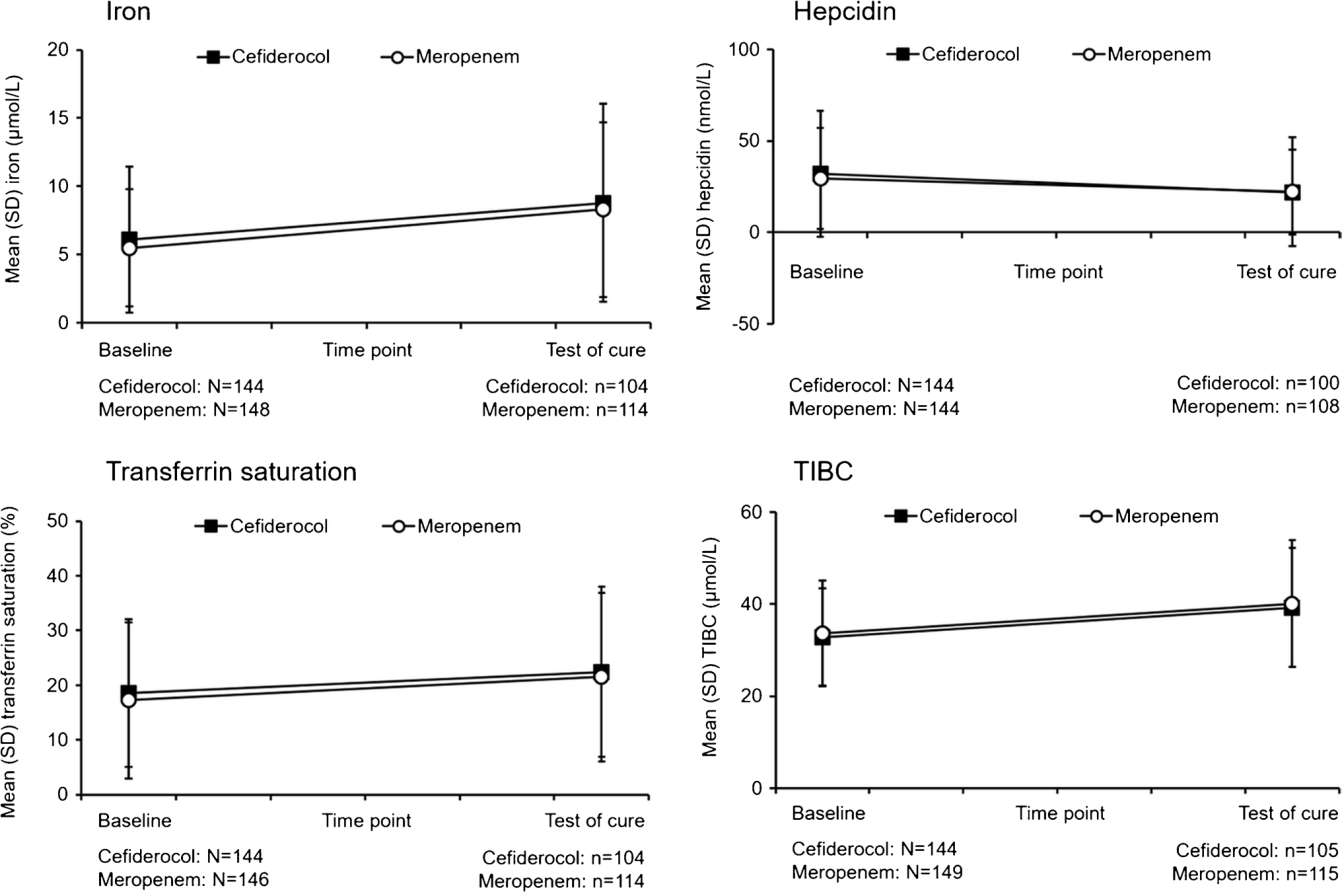
Changes in serum iron, hepcidin, transferrin saturation, and total iron binding capacity between baseline and test of cure in the overall safety population

Additionally, we evaluated changes from baseline to TOC in safety parameters specific for iron homeostasis, including hepcidin, total serum iron levels, transferrin saturation, and TIBC. The normal ranges for these parameters are shown in Supplementary Table 1. Among patients with measurements available at baseline and TOC, changes in levels of hepcidin, iron, transferrin saturation, and TIBC between the two time points were similar with cefiderocol and meropenem in the overall ITT/safety population (Fig. 2). The special laboratory findings showed that between baseline and TOC visits, hepcidin levels decreased, while total serum iron concentration, transferrin saturation, and TIBC increased in patients (Fig. 2).

### Blood transfusion and/or iron supplementation

A total of 37 patients in the cefiderocol arm and 27 patients in the meropenem arm received concomitant blood transfusion (cefiderocol arm *n* = 26; meropenem arm *n* = 17) and/or iron supplementation (cefiderocol arm *n* = 16; meropenem arm *n* = 11) by EOT; most of these patients had low baseline iron levels (cefiderocol arm 83.8%, meropenem arm 77.8%). We investigated the efficacy outcomes and safety parameters among this subgroup of patients compared with patients who did not receive any iron supplementation during treatment. Among patients receiving iron supplementation (i.e., blood, iron, or both) changes in the levels of hepcidin, iron, transferrin saturation, and TIBC between baseline and TOC were similar between treatment arms (Supplementary Fig. 1, 3, 5). In the subgroups of patients with no supplementation, changes in iron homeostasis parameters were also similar between treatment arms by TOC (Supplementary Fig. 2, 4, 6). However, patient numbers were small and therefore these results should be interpreted with caution.

At TOC, clinical cure rate was similar with cefiderocol for patients receiving and not receiving blood transfusion (65.4% vs 64.7%, respectively), but with meropenem clinical cure rate was 35.3% for those receiving blood transfusion compared with 70.8% for those not receiving blood transfusion. A similar trend was observed for microbiological eradication rates (Supplementary Table 2). Clinical cure rates were similar between treatment arms for patients receiving (cefiderocol arm 81.3%, meropenem arm 72.7%) and not receiving iron supplementation (cefiderocol arm 62.8%, meropenem arm 66.2%), and a numerical difference between treatment arms was seen in microbiological eradication rate for patients receiving iron supplementation (Supplementary Table 2). When patients received blood transfusion and/or iron supplementation, both clinical cure and microbiological eradication rates were numerically higher in the cefiderocol arm vs meropenem arm (Supplementary Table 2).

ACM rates with cefiderocol treatment were similar to or lower than those with meropenem treatment, both in patients receiving any concomitant supplementation of blood and/or iron supplementation by EOT, and in those not receiving any supplementation (Supplementary Table 3).

## Discussion

In this post hoc analysis of APEKS-NP, ACM rates at day 14, which was the primary endpoint in the study, were similar between cefiderocol and meropenem treatment arms in patients with low (12.3% and 11.6%, respectively) and normal (11.1% and 13.0% respectively) baseline iron levels. In the overall population, ACM at day 14 was 12.4% with cefiderocol treatment and 11.6% with meropenem treatment [13]. In the current analysis, both day 14 and day 28 ACM rates were aligned with mortality rates observed in the overall population [13]. There was a tendency for higher numerical clinical cure and microbiological eradication rates in patients with normal, compared with low, iron levels; however, given the relatively small number of patients with normal iron levels, the data should be interpreted with caution. The clinical cure and microbiological eradication rates at TOC in the current post hoc analysis were also generally aligned with the results of the overall mITT population [13].

We found that most patients in the APEKS-NP study had low baseline total serum iron levels, which might have been the result of elevated hepcidin levels due to acute infection or underlying comorbidities [5, 7, 8]. Baseline serum iron levels did not impact on the relative efficacy (ACM, clinical cure, and microbiological eradication) of cefiderocol vs meropenem in patients with Gram-negative nosocomial pneumonia. Overall, there was no difference between treatment arms in anemia-related adverse events, and hemoglobin levels remained stable with cefiderocol and meropenem treatment. Neither treatment led to clinically relevant anemia, despite the low serum iron levels in this critically ill patient population. These measured iron levels were total serum iron levels, which do not represent either total or free iron levels at the site of infection.

The baseline-to-TOC profiles for hepcidin, iron, transferrin saturation, and TIBC in serum were in line with what might be expected for infections that are resolving following antibiotic treatment. There was no difference in these profiles between cefiderocol (an iron-chelator cephalosporin) and meropenem (a carbapenem), indicating that cefiderocol did not impact on the iron-handling capacity of patients. These findings related to iron-homeostasis parameters are similar to those identified in other cefiderocol clinical studies (APEKS-cUTI and CREDIBLE-CR) [14]. Furthermore, these parameters showed improvements with cefiderocol treatment, regardless of whether patients received concomitant blood transfusion, iron supplementation, or both.

Low iron levels and anemia have frequently been described in critically ill patients and/or in those in intensive care units, and such patients frequently require red blood cell transfusion [15, 16]. The key factors contributing to these conditions are hemolysis, blood loss (e.g., due to extracorporeal circuit or surgery), and iatrogenic factors, including frequent blood sampling and hemodilution, as well as inflammation emerging during infection [16]. To compensate for anemia or iron deficiency, patients may be treated with blood transfusion and/or oral formulations of iron supplementation. In APEKS-NP, 21.5% of patients received a form of supplementation to correct for anemia or low iron levels. As cefiderocol is a siderophore, the use of iron supplementation might be anticipated to impact on its efficacy. We have not observed any negative impact on the baseline-to-TOC profiles for TIBC, iron, transferrin saturation, and hepcidin among patients receiving blood transfusion, iron supplementation, or both, or among those patients not receiving such supplementation. The clinical cure and microbiological eradication rates, and ACM were in general favorable or similar between cefiderocol and meropenem arms in the subgroup of patients receiving these supplementations.

These encouraging clinical results are supported by those obtained from neutropenic murine infection models, including an iron-overload condition, which showed that the efficacy of cefiderocol against Gram-negative infections was unlikely to be affected by iron supplementation [17, 18]. Cefiderocol plasma concentrations appeared to be unaltered by iron overload, being similar in iron-overloaded and control mice [17]. Following cefiderocol exposure, at concentrations representative of human dosing regimen, in mice inoculated with Enterobacterales, *A. baumannii*, or *P. aeruginosa*, there was no difference in the reduction of bacterial burden between mice with standard iron levels and those subjected to iron overload [18]. Our clinical data from the APEKS-NP study suggest that the efficacy and safety of cefiderocol were not influenced by low total serum iron levels in critically ill nosocomial pneumonia patients.

The siderophore receptors in Gram-negative bacteria that capture cefiderocol are upregulated in low iron conditions [19] suggesting that cefiderocol uptake would be enhanced in patients experiencing low iron levels; however, the efficacy of cefiderocol was not enhanced in these patients. One possible explanation for this finding might be a receptor-independent uptake of cefiderocol, with passive diffusion of the molecule through non-cognate receptors. A second possible explanation is that bacteria may be experiencing iron depletion due to low levels of bioavailable iron and are therefore in a low iron state, even when they infect patients with normal iron levels. In this case, the receptors that capture cefiderocol could be equally expressed between patients with both low iron and normal iron levels, and by extension, cefiderocol would be equally effective in both conditions.

## Conclusions

In conclusion, baseline iron level status and/or supplementation with blood and/or iron had no impact on the efficacy and safety of cefiderocol in critically ill nosocomial pneumonia patients, and there were no clinically relevant differences between the siderophore cefiderocol and the non-siderophore meropenem. The clinical utility of the siderophore cephalosporin cefiderocol has been demonstrated in patients with nosocomial pneumonia regardless of their iron homeostasis status or administration of iron supplementation.

## Supplementary Information

Below is the link to the electronic supplementary material.

Supplementary file1 (DOCX 2.36 MB)

## Data Availability

Data sharing related to the study at reasonable request can be made through Shionogi’s portal site (https://www.shionogi.com/global/en/company/policies/clinical-trial-data-transparency-policy.html).
