# Supplementary material for: Iron serum levels and iron homeostasis parameters in patients with nosocomial pneumonia treated with cefiderocol: post hoc analysis of the APEKS-NP study
Source: Eur J Clin Microbiol Infect Dis. 2022 Jan 13;41(3):467–76. doi: 10.1007/s10096-021-04399-9 (PMC8831352; doi:10.1007/s10096-021-04399-9)
Supplement: Supplementary file 1 — Supplementary file1 (DOCX 2.36 MB) [file 10096_2021_4399_MOESM1_ESM.docx]

**Iron serum levels and iron homeostasis parameters in patients with nosocomial pneumonia treated with cefiderocol: *post-hoc* analysis of the APEKS-NP study**

Eric P. Skaar,^a^ Roger Echols,^b^ Yuko Matsunaga,^c^ Anju Menon,^d^ Simon Portsmouth,^e*^

**Affiliations**

^a^ Vanderbilt University Medical Center, Department of Pathology, Microbiology, and Immunology, Nashville, TN 37232, USA.

^b^ Infectious Disease Drug Development Consulting, LLC, Easton, CT 06612, USA.

^c^ Shionogi Inc., Florham Park, NJ 07932, USA.

^d^ Shionogi Inc., Florham Park, NJ 07932, USA.

^e^ Shionogi Inc., Florham Park, NJ 07932, USA.

***Corresponding author:**

Simon Portsmouth

Shionogi Inc., Florham Park, 300 Campus Drive, 07932, NJ, USA

Tel: +1 862 777 4269

Email: [Simon.Portsmouth@shionogi.com](mailto:Simon.Portsmouth@shionogi.com)

**Supplementary Table 1** Laboratory test normal ranges

| **Laboratory Test** | **Unit** | **Lower Limit of Normal** | **Upper Limit of Normal** |
| --- | --- | --- | --- |
| Hepcidin | nmol/L | 0.53 | 14.84 |
| Iron – Male | µmol/L | 11 | 32 |
| Iron – Female | µmol/L | 7 | 31 |
| Total iron binding capacity | µmol/L | 45 | 81 |
| Transferrin | g/L | 2 | 3.6 |
| Transferrin saturation | % | 20 | 55 |

**Supplementary Table 2** Clinical and microbiological response rates at test of cure in patients with or without supplementation (blood transfusion, iron supplementation, or any) in the modified intention-to-treat population

|  | **With Blood Transfusion Only** | | | **Without Blood Transfusion** | | |
| --- | --- | --- | --- | --- | --- | --- |
|  | **Cefiderocol** | **Meropenem** | **Difference**  **(95% CI)** | **Cefiderocol** | **Meropenem** | **Difference**  **(95% CI)** |
| **Clinical response rate, n/N (%)** |  |  |  |  |  |  |
| Clinical cure | 17/26 (65.4) | 6/17 (35.3) | 30.1 (0.9, 59.3) | 77/119 (64.7) | 92/130 (70.8) | −6.1 (−17.7, 5.5) |
| Clinical failure | 5/26 (19.2) | 7/17 (41.2) |  | 22/119 (18.5) | 24/130 (18.5) |  |
| Indeterminate | 4/26 (15.4) | 4/17 (23.5) |  | 20/119 (16.8) | 14/130 (10.8) |  |
| **Microbiological response rate, n/N’ (%)** |  |  |  |  |  |  |
| Eradication | 11/24 (45.8) | 4/17 (23.5) | 22.3 (−6.1, 50.7) | 48/100 (48.0) | 57/110 (51.8) | −3.8 (−17.3, 9.7) |
| Persistence | 6/24 (25.0) | 5/17 (29.4) |  | 20/100 (20.0) | 22/110 (20.0) |  |
| Indeterminate | 7/24 (29.2) | 8/17 (47.1) |  | 32/100 (32.0) | 31/110 (28.2) |  |
|  | **With Iron Supplementation Only** | | | **Without Iron Supplementation** | | |
|  | **Cefiderocol** | **Meropenem** | **Difference**  **(95% CI)** | **Cefiderocol** | **Meropenem** | **Difference**  **(95% CI)** |
| **Clinical response rate, n/N (%)** |  |  |  |  |  |  |
| Clinical cure | 13/16 (81.3) | 8/11 (72.7) | 8.5 (−24.0, 41.1) | 81/129 (62.8) | 90/136 (66.2) | −3.4 (−14.9, 8.1) |
| Clinical failure | 3/16 (18.8) | 3/11 (27.3) |  | 24/129 (18.6) | 28/136 (20.6) |  |
| Indeterminate | 0/16 (0) | 0/11 (0) |  | 24/129 (18.6) | 18/136 (13.2) |  |
| **Microbiological response rate, n/N’ (%)** |  |  |  |  |  |  |
| Eradication | 9/12 (75.0) | 4/8 (50.0) | 25.0 | 50/112 (44.6) | 57/119 (47.9) | −3.3 (−16.1, 9.6) |
| Persistence | 0/12 (0) | 2/8 (25.0) |  | 26/112 (23.2) | 25/119 (21.0) |  |
| Indeterminate | 3/12 (25.0) | 2/8 (25.0) |  | 36/112 (32.1) | 37/119 (31.1) |  |
|  | **With Blood Transfusion and/or Iron Supplementation** | | | **Without Any Supplementation** | | |
|  | **Cefiderocol** | **Meropenem** | **Difference**  **(95% CI)** | **Cefiderocol** | **Meropenem** | **Difference**  **(95% CI)** |
| **Clinical response rate, n/N (%)** |  |  |  |  |  |  |
| Clinical cure | 26/37 (70.3) | 13/27 (48.1) | 22.1 (−1.8, 46.0) | 68/108 (63.0) | 85/120 (70.8) | −7.9 (−20.1, 4.3) |
| Clinical failure | 7/37 (18.9) | 10/27 (37.0) |  | 20/108 (18.5) | 21/120 (17.5) |  |
| Indeterminate | 4/37 (10.8) | 4/27 (14.8) |  | 20/108 (18.5) | 14/120 (11.7) |  |
| **Microbiological response rate, n/N’ (%)** |  |  |  |  |  |  |
| Eradication | 18/32 (56.3) | 8/24 (33.3) | 22.9 (−2.6, 48.4) | 41/92 (44.6) | 53/103 (51.5) | −6.9 (−20.9, 7.1) |
| Persistence | 6/32 (18.8) | 7/24 (29.2) |  | 20/92 (21.7) | 20/103 (19.4) |  |
| Indeterminate | 8/32 (25.0) | 9/24 (37.5) |  | 31/92 (33.7) | 30/103 (29.1) |  |

All patients in the overall mITT population are included regardless of their baseline iron level status.

Abbreviations: CI: confidence interval; ITT: intention-to-treat; N: patients in the modified ITT population receiving the corresponding iron supplementation; N’: patients with non-missing baseline pathogens.

**Supplementary Table 3** All-cause mortality rates in patients with or without supplementation (blood transfusion, iron supplementation, or both) in the modified intention-to-treat population

|  | **With Blood Transfusion Only** | | | **Without Blood Transfusion** | | |
| --- | --- | --- | --- | --- | --- | --- |
| **ACM rate, n/N (%)** | **Cefiderocol** | **Meropenem** | **Difference**  **(95% CI)** | **Cefiderocol** | **Meropenem** | **Difference**  **(95% CI)** |
| Day 14 | 5/26 (19.2) | 6/17 (35.3) | −16.1 (−43.4, 11.2) | 13/119 (10.9) | 11/129 (8.5) | 2.4 (−5.0, 9.8) |
| Day 28 | 8/25 (32.0) | 7/17 (41.2) | −9.2 (−38.9, 20.5) | 22/118 (18.6) | 23/129 (17.8) | 0.8 (−8.8, 10.5) |
| EOS | 10/25 (40.0) | 8/17 (47.1) | −7.1 (−37.6, 23.5) | 28/117 (23.9) | 26/129 (20.2) | 3.8 (−6.6, 14.2) |
|  | **With Iron Supplementation Only** | | | **Without Iron Supplementation** | | |
| **ACM rate, n/N (%)** | **Cefiderocol** | **Meropenem** | **Difference**  **(95% CI)** | **Cefiderocol** | **Meropenem** | **Difference**  **(95% CI)** |
| Day 14 | 0/16 (0) | 1/11 (9.1) | −9.1 (−26.1, 7.9) | 18/129 (14.0) | 16/135 (11.9) | 2.1 (−6.0, 10.2) |
| Day 28 | 2/16 (12.5) | 3/11 (27.3) | −14.8 (−45.7, 16.1) | 28/127 (22.0) | 27/135 (20.0) | 2.0 (−7.8, 11.9) |
| EOS | 3/16 (18.8) | 3/11 (27.3) | −8.5 (−41.1, 24.0) | 35/126 (27.8) | 31/135 (23.0) | 4.8 (−5.7, 15.4) |
|  | **With Blood Transfusion and/or Iron Supplementation** | | | **Without Any Supplementation** | | |
| **ACM rate, n/N (%)** | **Cefiderocol** | **Meropenem** | **Difference**  **(95% CI)** | **Cefiderocol** | **Meropenem** | **Difference**  **(95% CI)** |
| Day 14 | 5/37 (13.5) | 7/27 (25.9) | −12.4 (−32.3, 7.5) | 13/108 (12.0) | 10/119 (8.4) | 3.6 (−4.3, 11.5) |
| Day 28 | 9/36 (25.0) | 10/27 (37.0) | −12.0 (−35.1, 11.0) | 21/107 (19.6) | 20/119 (16.8) | 2.8 (−7.3, 12.9) |
| EOS | 12/36 (33.3) | 11/27 (40.7) | −7.4 (−31.5, 16.7) | 26/106 (24.5) | 23/119 (19.3) | 5.2 (−5.6, 16.0) |

All patients in the overall mITT population are included regardless of their baseline iron level status.

Abbreviations: ACM: all-cause mortality; EOS: end of study; CI: confidence interval; ITT: intention-to-treat; N: patients with known vital status in the modified ITT population receiving the corresponding iron supplementation.**Supplementary Fig 1** Changes in serum iron homeostasis parameters between baseline and test of cure in patients with blood transfusion in the safety population


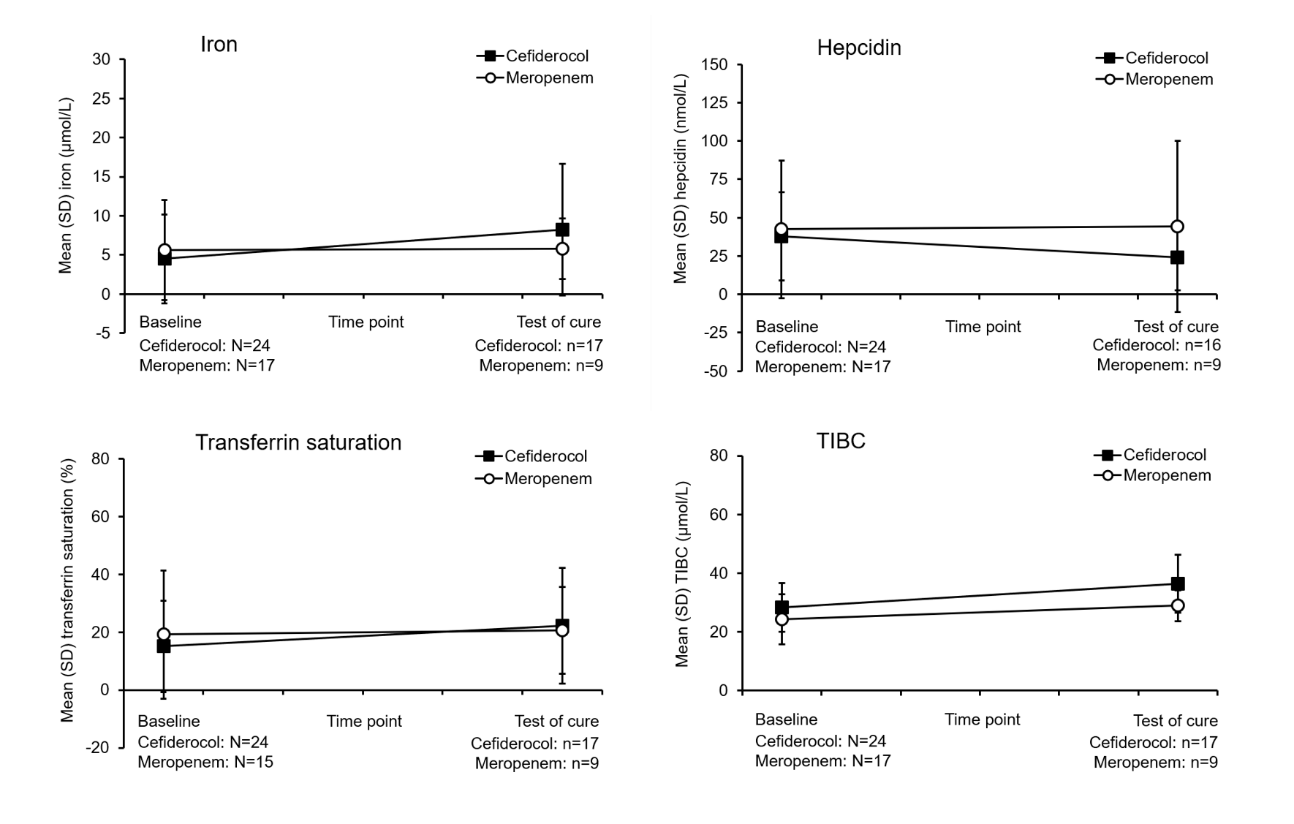


Abbreviations: SD: standard deviation; TIBC: total iron binding capacity.

**Supplementary Fig 2** Changes in serum iron homeostasis parameters between baseline and test of cure in patients without blood transfusion in the safety population

**
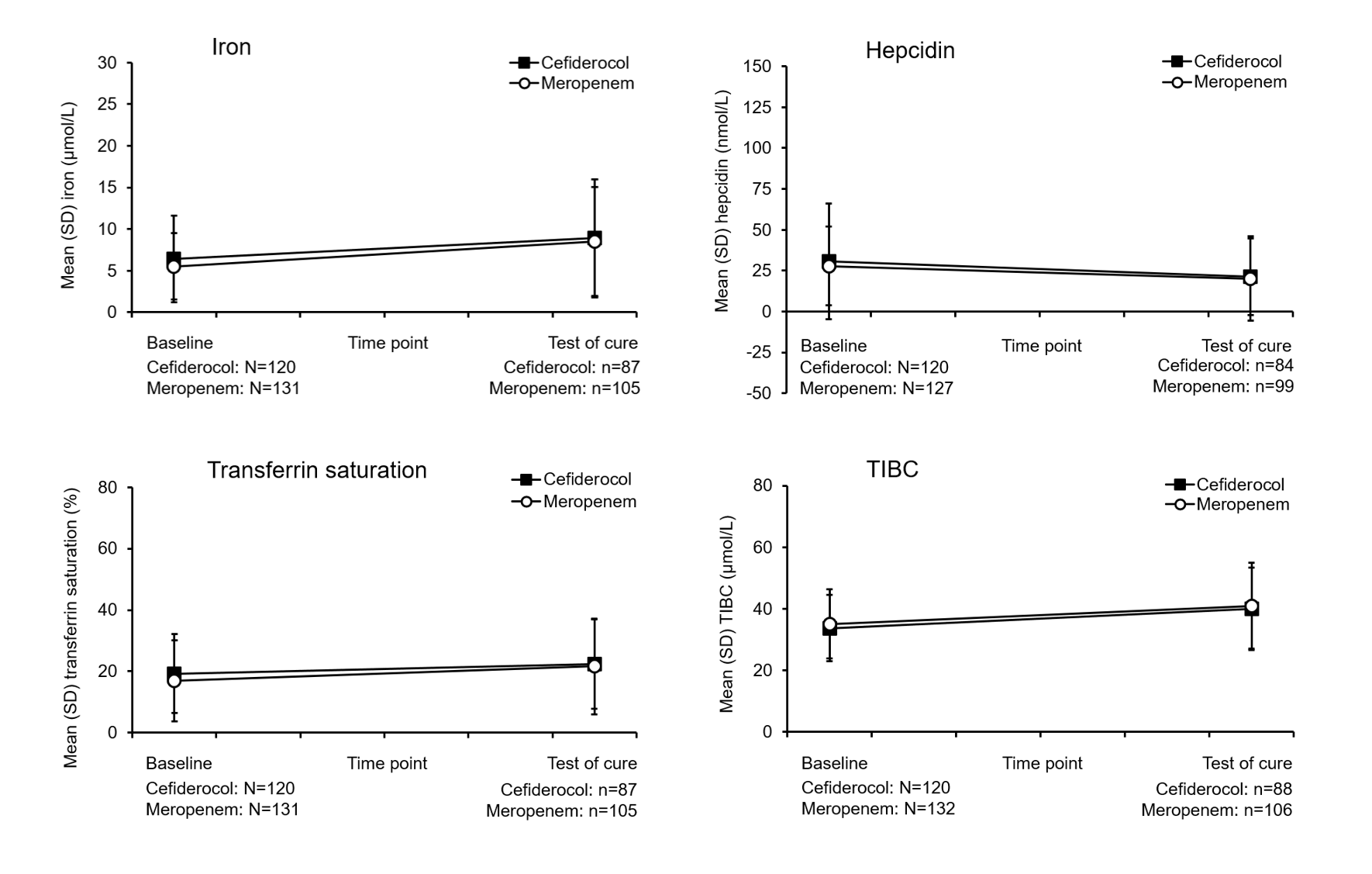
**

Abbreviations: SD: standard deviation; TIBC: total iron binding capacity.

**Supplementary Fig 3** Changes in serum iron homeostasis parameters between baseline and test of cure in patients with iron supplementation in the safety population


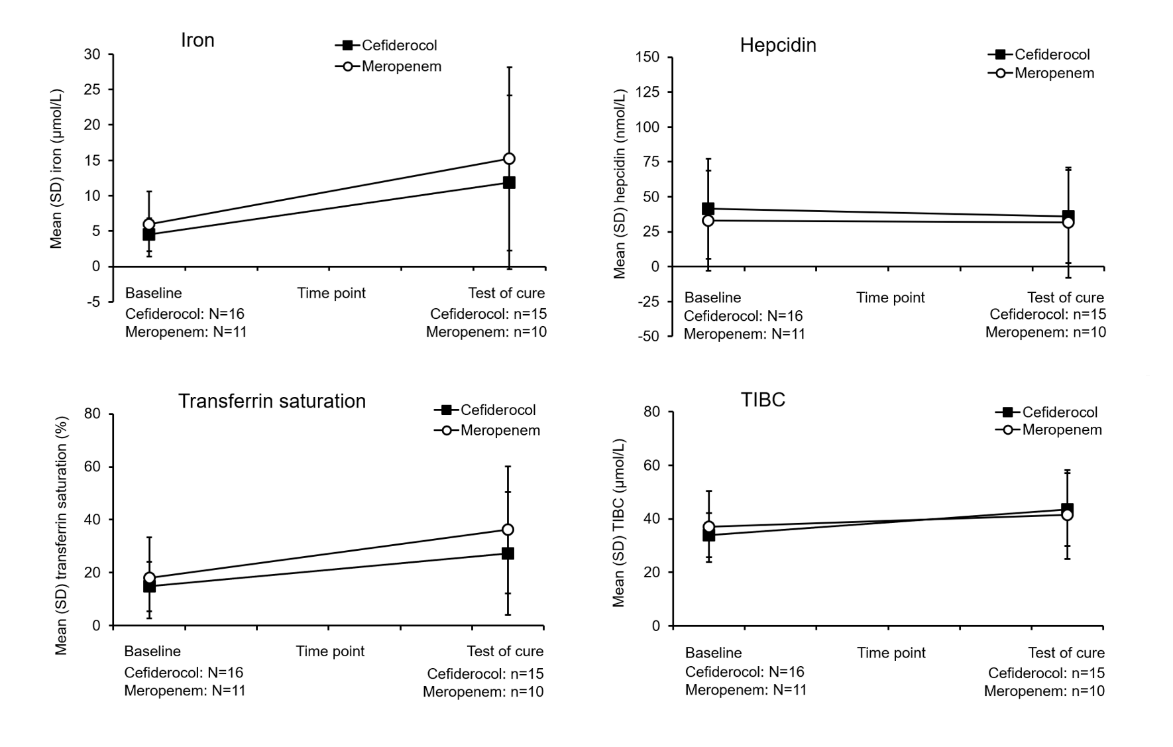


Abbreviations: SD: standard deviation; TIBC: total iron binding capacity.

**Supplementary Fig 4** Changes in serum iron homeostasis parameters between baseline and test of cure in patients without iron supplementation in the safety population


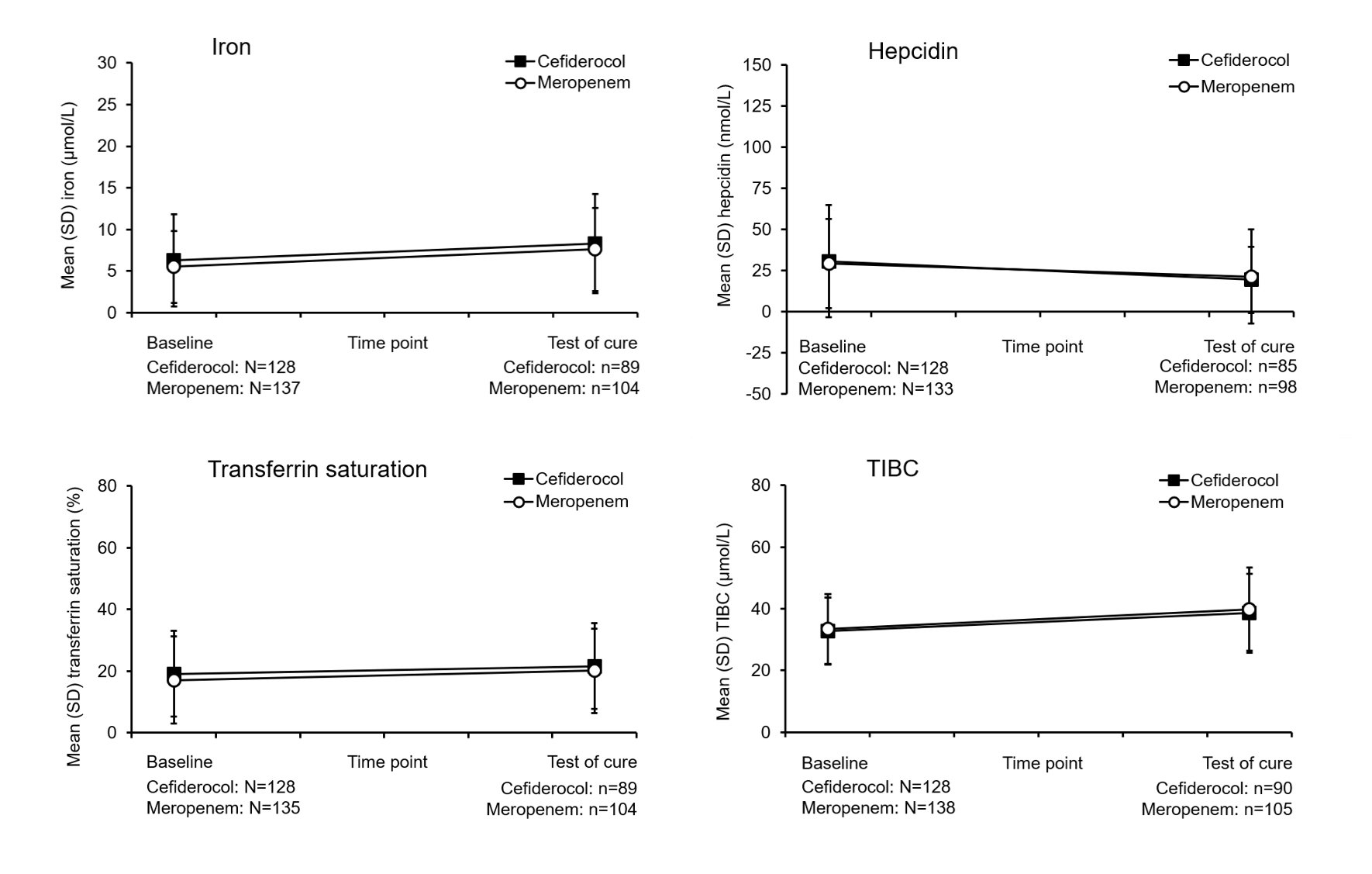


Abbreviations: SD: standard deviation; TIBC: total iron binding capacity.

**Supplementary Fig 5** Changes in serum iron homeostasis parameters between baseline and test of cure in patients with blood transfusion and/or iron supplementation in the safety population

**
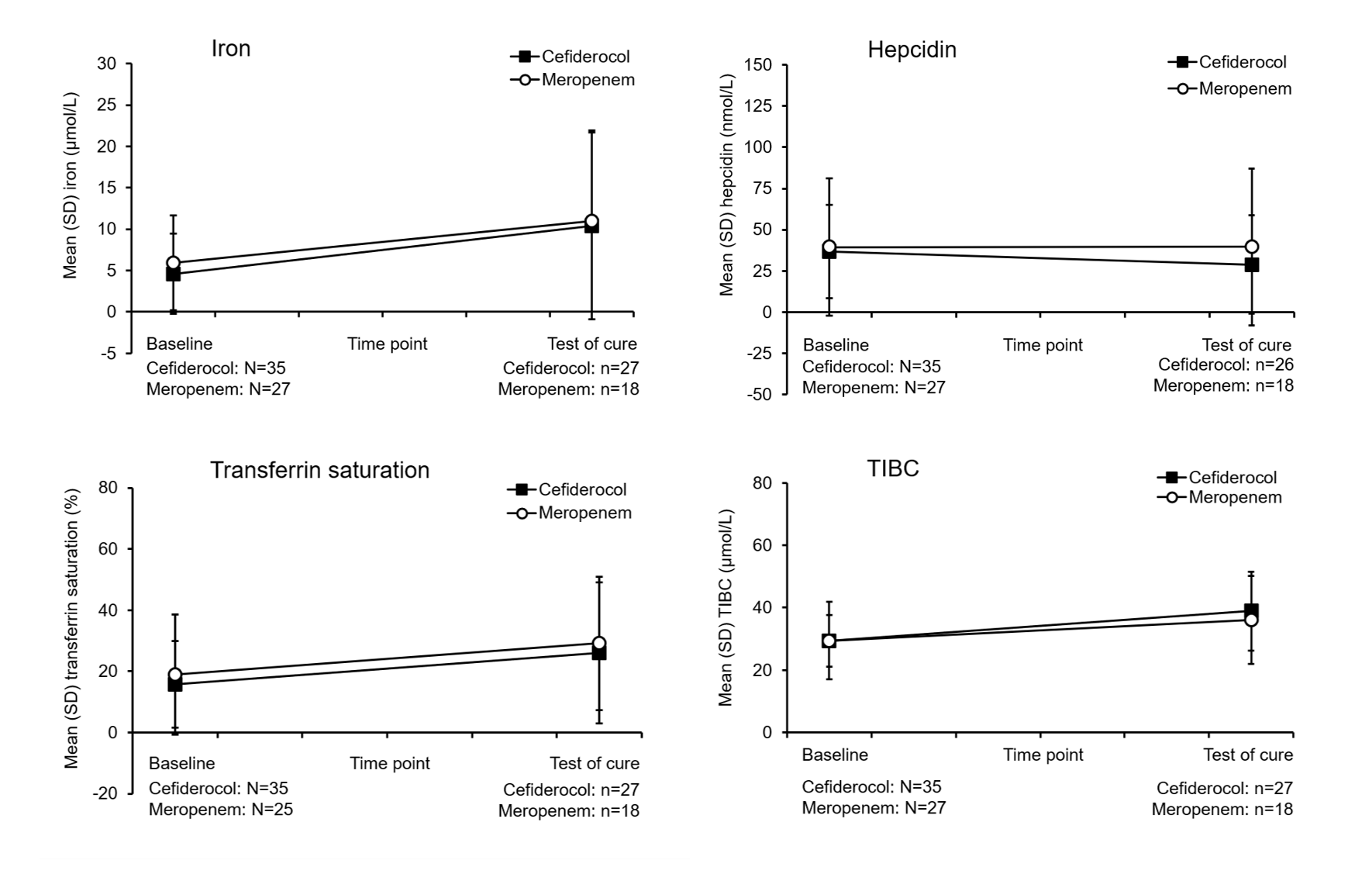
**

Abbreviations: SD: standard deviation; TIBC, total iron binding capacity.

**Supplementary Fig 6** Changes in serum iron homeostasis parameters between baseline and test of cure in patients without blood transfusion and/or iron supplementation in the safety population

**
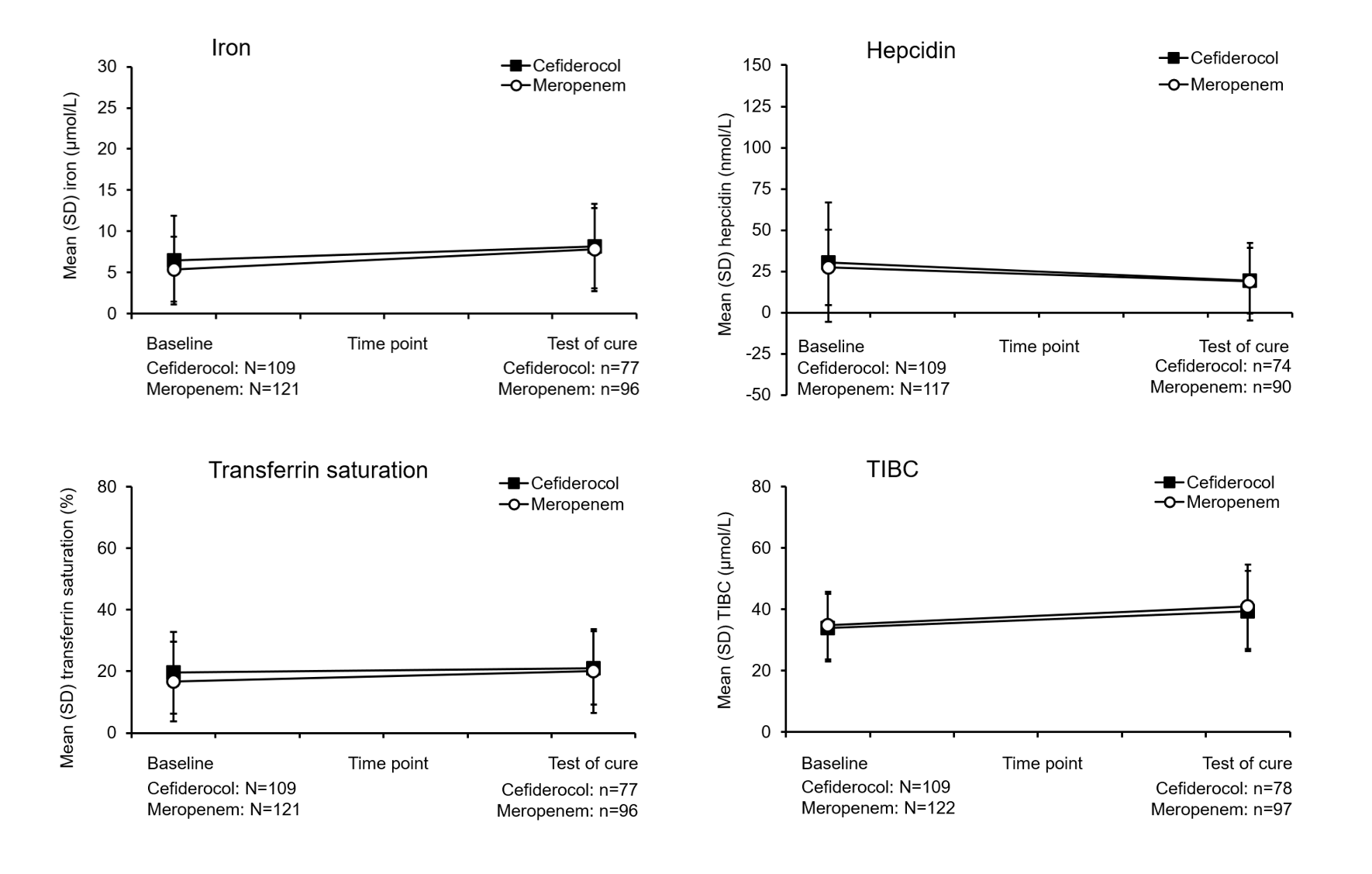
**

Abbreviations: SD: standard deviation; TIBC: total iron binding capacity.
